# Effect of Strain Rate on Mechanical Deformation Behavior in CuZr Metallic Glass

**DOI:** 10.3390/ma17112507

**Published:** 2024-05-23

**Authors:** Beibei Fan, Maozhi Li

**Affiliations:** 1 Beijing Key Laboratory of Opto-Electronic Functional Materials & Micro-Nano Devices, Department of Physics, Renmin University of China, Beijing 100872, China; 2015101971@ruc.edu.cn; 2 Key Laboratory of Quantum State Construction and Manipulation (Ministry of Education), Renmin University of China, Beijing 100872, China

**Keywords:** metallic glass, deformation behavior, strain rate, mechanical response, molecular simulation

## Abstract

Tensile tests were performed on Cu_64_Zr_36_ metallic glass at strain rates of 10^7^/s, 10^8^/s, and 10^9^/s via classical molecular dynamics simulations to explore the underlying mechanism by which strain rate affects deformation behavior. It was found that strain rate has a great impact on the deformation behavior of metallic glass. The higher the strain rate is, the larger the yield strength. We also found that the strain rate changes the atomic structure evolution during deformation, but that the difference in the atomic structure evolution induced by different strain rates is not significant. However, the mechanical response under deformation conditions is found to be significantly different with the change in strain rate. The average von Mises strain of a system in the case of 10^7^/s is much larger than that of 10^9^/s. In contrast, more atoms tend to participate in deformation with increasing strain rate, indicating that the strain localization degree is more significant in cases of lower strain rates. Therefore, increasing the strain rate reduces the degree of deformation heterogeneity, leading to an increase in yield strength. Further analysis shows that the structural features of atomic clusters faded out during deformation as the strain rate increased, benefiting more homogeneous deformation behavior. Our findings provide more useful insights into the deformation mechanisms of metallic glass.

## 1. Introduction

Metallic glasses exhibit unique mechanical properties compared to their crystalline counterparts, such as a high limit of elastic deformation and extraordinary strength [1,2,3], making them potentially useful for application in different industries, as well as in academic uses and advanced material applications [1,2,3,4]. However, the catastrophic failure of metallic glasses under deformation at room temperature severely hampers their applications [5,6]. The underlying mechanism responsible for the deformation behavior remains unclear.

It has been found that metallic glasses exhibit quite different deformation behaviors under loading at different strain rates [4,5,6,7,8,9,10], and a lot of effort has been devoted to tuning the mechanical behavior of metallic glasses by changing the strain rate [11,12,13,14,15,16,17,18,19,20]. For example, yield strength shows a positive dependence on the strain rate from 10^−4^/s to 10^−2^/s in Ti-based metallic glasses under compression conditions [11]. Nanoindentation experiments with different loading rates also found that the strength of different metallic glasses increases to various degrees with the increase in strain rate in the same range from 0.33 mN/s to 264.0 mN/s [12]. In addition, some molecular dynamics simulations also showed that the yield strength increases with the increasing of the strain rate from 10^7^/s to 10^9^/s in CuZr metallic glasses under tension [13,14,15]. However, the dynamic compression tests used in experiments show that the yield strength of Zr-based metallic glass decreases with increasing strain rates from 10^−4^/s to 10^3^/s [16,17]. In contrast, tensile tests for Pd_40_Ni_40_P_20_ metallic glass show that the yield strength is nearly independent of the strain rate from 10^−4^/s to 10^3^/s [18]. Similar behavior was also observed in Zr-based metallic glasses in compressive and tensile tests across a range of strain rates from 10^−5^/s to 10^−1^/s [19,20]. Therefore, strain rate has a great impact on the mechanical deformation behavior of metallic glasses upon loading. While high strain rates result in an increase in yield strength, very low strain rates often lead to a decrease in the yield strength of metallic glasses during deformation. Additionally, it is found that under compression tests, the yield strength of different compositions may increase [11], decrease [16,17], or remain unchanged [19,20] with an increase in strain rate. Similarly, under tensile tests, the yield strength of different compositions can also exhibit different strain-rate-dependent behaviors [13,14,15,18,19,20]. Furthermore, within the same composition, tension or compression may affect the plasticity and yield strength of the system but do not influence the strain-rate-dependent behavior of the yield strength [20].

So far, numerous studies have been conducted to explore the mechanisms by which the strain rate affects the deformation behavior of Ti-based, Zr-based, and Pd-based metallic glasses [11,12,13,14,15,16,17,18,19,20]. It was experimentally revealed that shear band density is reduced as the strain rate increases from 10^−4^/s to 10^−2^/s, which causes an increase in the fracture stress in Ti-based metallic glasses [11]. A similar reduction in shear band density is also observed with the change in strain rate from 10^−3^/s to 10^3^/s in Pd-based metallic glasses, whereas the yield stress changes little with strain rate [16]. An increase is also observed in yield strength when the strain rate increases from 2 × 10^−4^/s to 7.5 × 10^−3^/s in Zr_55.__7_Cu_22.__4_Al_14.__7_Ni_7.__2_ metallic glass at high temperatures, which is attributed to the migration of more atoms and the generation of more free volume, which is induced by higher stress during loading with higher strain rates [21]. Recent molecular dynamic simulations found that a higher strain rate delays mechanical transformations in regions with higher local five-fold symmetry, which retards the propagation of shear band and promotes more uniform deformation in Cu_50_Zr_50_ metallic glass [13]. However, the degree of five-fold local symmetry is nearly the same under the same yield strain for different strain rates from 4 × 10^7^/s to 4 × 10^9^/s [13]. In Mg-Al systems, the increase in the yield strength under high strain rates is because the rapid application of strain does not allow for sufficient time for atoms to rearrange, hampering stress relaxation [22]. Therefore, the underlying mechanism of strain rate on deformation behavior in metallic glasses upon loading is still hotly debated.

In this study, we performed tensile deformation for Cu_64_Zr_36_ metallic glass with strain rates of 10^7^/s, 10^8^/s, and 10^9^/s via classical molecular dynamics simulations to investigate the effect of strain rate on the mechanical deformation behavior and its underlying mechanisms. It is found that yield strength increases with increasing strain rate. It is also found that the atomic structure evolution under deformation conditions exhibits different behaviors, but that the difference induced by different strain rates is not significant. Further analysis reveals that the mechanical response of metallic glass is significantly different under deformation with different strain rates. While the average von Mises strain decreases with increasing strain rate, the number of atoms participating in deformation increases, leading to a change from a higher degree of strain localization in the case of 10^7^/s to less localized deformation in the case of 10^9^/s. These findings indicate that more localized and heterogeneous plastic deformation transforms into less localized and relatively homogeneous deformation in Cu_64_Zr_36_ metallic glass as the strain rate increases from 10^7^/s to 10^9^/s, leading to different deformation behaviors and an increase in yield strength.

## 2. Model and Method

Classical molecular dynamics simulations were performed using the Large-scale Atomic/Molecular Massively Parallel Simulator (LAMMPS) package version 16 November 2017 [23] for Cu_64_Zr_36_ metallic glass, with the interatomic interactions described using a realistic embedded-atom method to assess potential [24]. The system contains 40,000 atoms in a cubic box, with periodic boundary conditions (PBC) applied in three directions. The initial configuration was first fully equilibrated at 2000 K in an isothermal–isobaric (NPT) ensemble, and then quenched down to 300 K with a cooling rate of 10^11^ K/s to generate glassy samples. During quenching, the box size was adjusted to give zero pressure in the NPT ensemble. The glassy samples at 300 K were then relaxed for 3 ns in the NPT ensemble before loading. Then, tensile deformation was applied to the sample at 300 K along the Z axis and PBC was applied in three directions. Here, strain rates of 10^7^/s, 10^8^/s, and 10^9^/s were applied in MD simulations because simulations with lower strain rates struggle to be accessible. Previous studies show that icosahedral clusters play important roles in the dynamical and mechanical properties of CuZr metallic liquids and glasses [25,26,27,28]. In order to elucidate the role of icosahedral clusters in the mechanical deformation behavior induced by different strain rates, Cu_64_Zr_36_ metallic glass with a higher population of icosahedral clusters was chosen in our work.

## 3. Results and Discussions

### 3.1. Atomic Structural Evolution with Different Strain Rates

**Figure 1:**
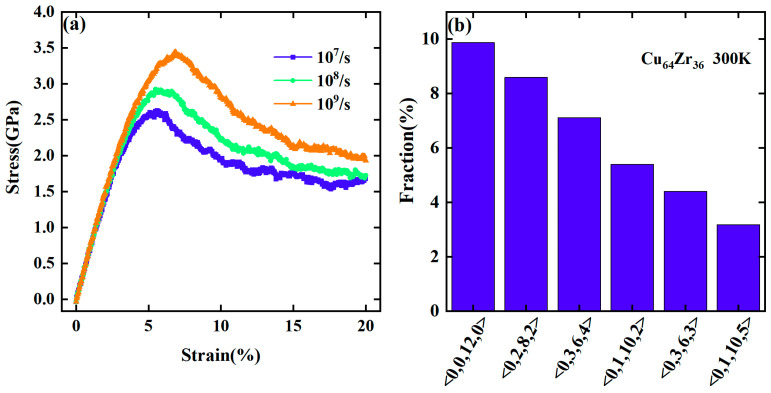
(**a**) Strain–stress curves under uniaxial tensile deformation of Cu_64_Zr_36_ metallic glassy samples with strain rates of 10^7^/s, 10^8^/s and 10^9^/s, respectively. (**b**) Fraction of six major populated atomic clusters in Cu_64_Zr_36_ metallic glassy samples at 300 K with cooling rate of 10^11^ K/s.

Figure 1a shows the typical strain–stress curves for up to 20% strain in metallic glassy samples in the tensile deformation process at 300 K, with strain rates of 10^7^/s, 10^8^/s, and 10^9^/s, respectively. It can be seen that these curves collapse together in the elastic regime, indicating that the strain rate has little impact on the deformation behavior of metallic glasses in this regime. However, the yield strength significantly increases with the increasing strain rate, indicating that strain rate significantly affects the yielding behavior of metallic glasses. This is consistent with previous studies [22,29,30].

**Figure 2:**
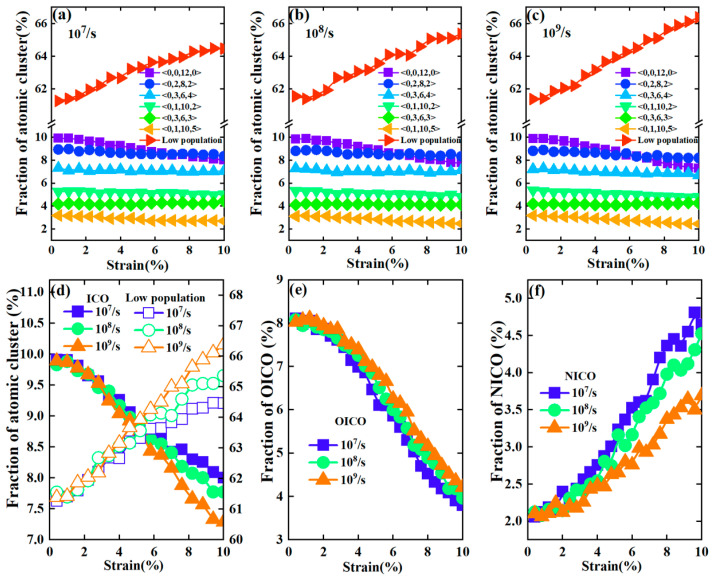
The fraction evolution of the major populated atomic clusters and low-population clusters of systems under deformation with strain rates of 10^7^/s (**a**), 10^8^/s (**b**), and 10^9^/s (**c**), respectively. The fraction of icosahedral clusters and low-population clusters (**d**), OICO (**e**), and NICO (**f**) during the deformation of Cu_64_Zr_36_ metallic glass with three different strain rates. The icosahedral clusters in the initial configurations and those that were newly formed during deformation were denoted as OICO and NICO, respectively. The fraction of ICO, OICO, and NICO was calculated as the total number of ICO, OICO, and NICO divided by the total number of atoms, respectively.

To understand the mechanism behind the impact of strain rate on yielding behavior, we analyzed the atomic structure evolution of the glassy sample during deformation. Voronoi tessellation was employed to characterize the local atomic structures in terms of the Voronoi index and its values of <n_3_, n_4_, n_5_, n_6_>, where n*_i_* is the number of *i*-edged faces in a Voronoi polyhedron [31]. Figure 1b shows six major populated atomic clusters whose fraction is larger than 3% in the initial atomic configurations of metallic glassy samples at 300 K. The fraction was calculated by dividing the number of each type of cluster by the total number of atoms. The remaining types of clusters were considered to be low-population ones. We analyzed the fraction evolution of these types of atomic clusters, as well as those of low-population ones, during deformation at various strain rates. As shown in Figure 2a–c, the fraction of icosahedral clusters of <0, 0, 12, 0> decreases significantly compared to other five major populated clusters, the fraction of which changes little during deformation. In contrast, the fraction of the low-populated clusters shows significant increases during deformation and increases more with the increasing strain rate.

Figure 2d shows closer comparisons of fraction evolution for <0, 0, 12, 0> and low-population clusters in terms of deformation with different strain rates. It can be seen that the strain rate does change the atomic evolution behavior observed during deformation. However, the difference in the fraction evolution under different strain rates is very small. We also analyzed the fraction evolution of icosahedral clusters in the initial configurations and those newly formed during deformation, denoted as OICO and NICO, respectively. Figure 2e,f show the changes in the fraction of OICO and NICO during deformation in three different strain rates, respectively. It can be seen from Figure 2e that the fraction of OICO in the initial configuration decreases faster with a strain rate of 10^7^/s, but that the decrease becomes slower as the strain rate increases. However, the fraction of NICO increases more significantly with a strain rate of 10^7^/s, but the increase becomes slower with an increasing strain rate, as shown in Figure 2f. Therefore, upon loading, some OICO clusters are destroyed and transformed into other types of clusters; meanwhile, other types of clusters are transforming into NICO ones. The opposite effect of the OICO and NICO clusters during deformation results in small differences in the total fraction of icosahedral clusters for different strain rates.

**Figure 3:**
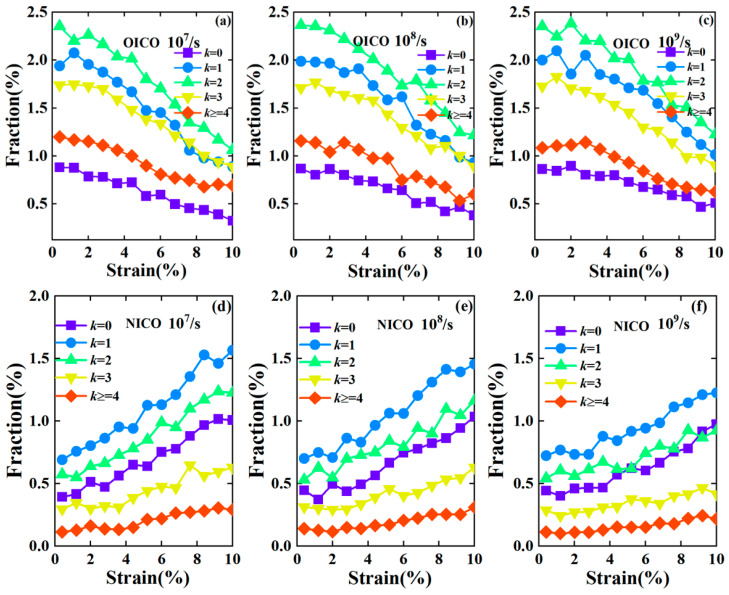
The fraction evolution of node degree in the icosahedral network in the initial structures of each Cu_64_Zr_36_ glassy sample under deformations with strain rates of 10^7^/s (**a**), 10^8^/s (**b**), 10^9^/s (**c**). The fraction evolution of the newly formed icosahedral clusters (NICO) under deformation with strain rates of 10^7^/s (**d**), 10^8^/s (**e**), 10^9^/s (**f**).

We also analyzed the network formed by the interpenetrating icosahedral clusters [25,32] to obtain more insights into the atomic structure evolution during deformation at different strain rates. The central atoms of icosahedral clusters were regarded as network nodes, and the number of icosahedral clusters connected to a node defined the degree of the node, *k*. Figure 3 shows the fraction evolution of the node degree in OICO and NICO networks during deformation at different strain rates, respectively. This fraction is obtained by dividing the number of node degrees by the total number of atoms. It can be seen from Figure 3a that the fraction of different node degrees in the OICO network decreases faster with a strain rate of 10^7^/s, but this becomes slower with an increase in strain rate. However, the fraction of node degree in a NICO network increases more significantly with a strain rate of 10^7^/s, but increases slower with an increasing strain rate, as shown in Figure 3d–f. It can be seen that the difference in the fraction evolution in terms of node degree in OICO and NICO networks under different strain rates is also small.

### 3.2. Mechanical Response to Different Strain Rates

**Figure 4:**
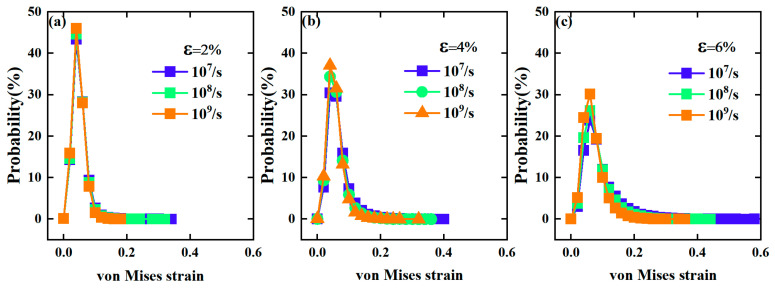
The probability distribution of the atomic von Mises strain at the strain of 2% (**a**), 4% (**b**) and 6% (**c**) under deformation with different strain rates, respectively.

Based on the above analysis, it can be seen that different strain rates may lead to different atomic structure evolution behaviors during deformation. However, the difference in the atomic structure evolution induced by different strain rates is not significant, and might not be responsible for the significant difference in yielding behavior caused by different strain rates. Next, we analyzed the mechanical response of metallic glasses under deformation with different strain rates. Here, the local shear strain invariant, i.e., von Mises, strain $\eta^{Mises}$ [33] was employed to characterize the mechanical response of each atom in the samples. This was defined in the following manner:

$$\eta^{Mises}=\sqrt{\frac{1}{2}Tr{\left(\eta -\eta_{m}I\right)}^{2}}$$

where η and $\eta_{m}$ are the local Lagrangian strain and local hydrostatic strain for that atom, respectively. To calculate $\eta^{Mises}$ of each atom, the initial configuration is often used as the reference configuration. The value of $\eta^{Mises}$ reveals the scale of the local shear strain for an atom during deformation. Figure 4 compares the probability distribution of von Mises strains in the samples under deformation with three strain rates obtained for different strains, respectively. At the same deformation strain, the distributions of von Mises strains for different strain rates exhibit quite similar behaviors, showing a peak with a long tail of larger values. However, the peak height increases a little, and the tail distribution becomes shorter, as the strain rate increases. This indicates that atoms experience larger von Mises strains during deformation with a lower strain rate, i.e., 10^7^/s. Thus, strain rate may have a significant impact on the mechanical response of metallic glasses.

**Figure 5:**
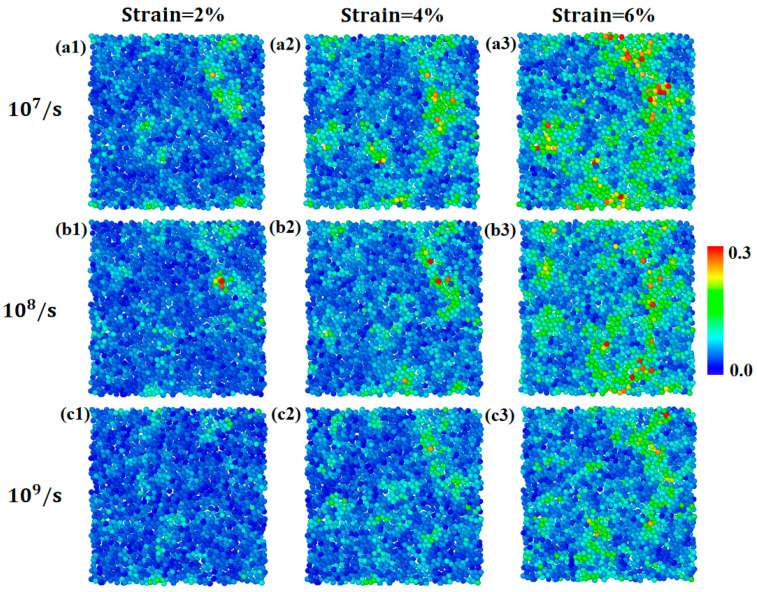
The slice showing the spatial distribution of the atomic von Mises strain at the strain of 2%, 4%, and 6% for strain rates of $10^{7}/s\;$ (**a1**–**a3**), $10^{8}/s\;$ (**b1**–**b3**) and $10^{9}/s$ (**c1**–**c3**), respectively. The slice was taken in the middle of the sample along the Z direction with a thickness of 3.8Å.

The impact of strain rate on the mechanical response of the metallic glassy samples is illustrated more clearly in Figure 5, which shows snapshots of the spatial distribution of the atomic von Mises strain at strains of 2%, 4%, and 6%, with strain rates of 10^7^/s, 10^8^/s, and 10^9^/s, respectively. The slice was taken from the middle of the sample along the Z direction with a thickness of 3.8Å. The results for the entire sample are shown in Supplementary Figure S1. It can be seen that the spatial distribution of atomic strain exhibits different characteristics under different strain rates. At a strain of 2%, although the strain–stress curves collapse together, showing almost no difference, larger atomic von Mises strains are distributed in the sample with a strain rate of 10^7^/s, while the distribution becomes more homogeneous in the sample as the strain rate increases to 10^9^/s. With increasing strain, more atoms experience much larger von Mises strains. The sample with a strain rate of 10^9^/s also exhibits less homogeneous distribution at a strain rate of 4%, compared to that at strain of 2%, while the distribution in the case of 10^7^/s becomes more heterogeneous than that seen at a strain of 4%. The above findings indicate that mechanical deformation becomes more homogeneous as strain rate increases.

**Figure 6:**
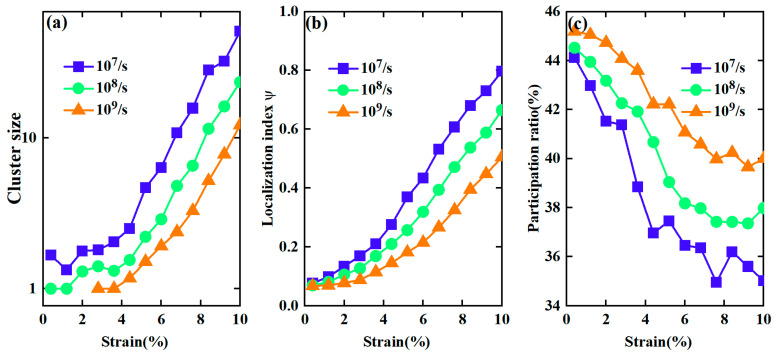
The average cluster size formed by the nearest neighboring atoms with von Mises strains larger than 0.2 (**a**); strain localization parameter ψ as a function of strain during deformation with various strain rates (**b**); the average participation ratio (PR) as a function of strain with a strain interval of 1% (**c**).

To perform a quantitative comparison of the distribution of atomic von Mises strains induced by different strain rates, we analyzed the cluster size formed by the nearest neighboring atoms with $\eta_{i}^{Mises}\;$ larger than a threshold of 0.2, which may be used to quantify the degree of mechanical heterogeneity of metallic glasses under deformation conditions. First, we identified all atoms with a von Mises strain larger than 0.2 and used these atoms as centers to find their nearest neighbors with $\eta_{i}^{Mises}>0.2$. Subsequently, we used these nearest neighbor atoms as new centers to search for all their nearest neighbors with $\eta_{i}^{Mises}>0.2$. This process was repeated until no further nearest neighbors with $\eta_{i}^{Mises}>0.2$ were found, and a cluster was identified. The total number of atoms in the cluster defined its size. The threshold values of 0.1 and 0.3 were also tested, and similar results were obtained (see Supplementary Figure S2). Figure 6a shows the evolution of the average cluster size during deformation with different strain rates, respectively. The average cluster size increases with strain, which is more drastic in the case of 10^7^/s. In contrast, the cluster size is much smaller in the case of 10^9^/s and the increase in the size of the strain is relatively slower. This also indicates that the mechanical response of metallic glasses to strain rate is quite different, and mechanical deformation becomes more homogeneous with an increasing strain rate. On the other hand, the above results imply that the mechanical deformation at a strain rate of 10^7^/s should be more localized. Therefore, we also characterized the localization degree by the localization parameter, defined as follows [34,35]:

$$\psi =\sqrt{\frac{1}{N}\sum_{i=1}^{N}{\left(\eta_{i}^{Mises}-\eta_{ave}^{Mises}\right)}^{2}}$$

where $\eta_{ave}^{Mises}$ is the average of von Mises strain of all atoms and N is the total number of atoms. This parameter evaluates the difference in strain distribution based on homogeneous behavior [35]. Larger values of ψ indicate large variation in the local atomic strain and a more localized deformation mode. Figure 6b shows the change in ψ values under deformation with different strain rates, respectively. It can be seen that the value of ψ increases with increasing strain for three different strain rates, indicating a tendency towards the strain localization of metallic glasses under deformation conditions. However, the ψ value increases much faster in the case of 10^7^/s, implying a higher degree of strain localization with lower strain rates. A higher degree of localization may lead to an earlier sample yield and reduce intrinsic plasticity during deformation [35].

To obtain more insight into the mechanical response of the glassy samples to the strain rate, we also calculated the deformation participation ratio (PR) [36] to characterize the deformation propensity of atoms under loading conditions. PR is defined as the fraction of atoms with a von Mises strain larger than the average value. A larger PR indicates that more atoms tend to participate in deformation. Therefore, it is expected that the PR will be small if a system undergoes highly localized deformation. Figure 6c shows PR as a function of strain in the glassy samples with different strain rates. Here, the configurations at a strain of 1% less than of the present ones were used as references for the calculation of $\eta_{i}^{Mises}$. As expected, the PR values exhibited an obvious decreasing trend as strain increased, especially for strain rate of 10^7^/s, indicating that fewer atoms were involved in deformation as strain increases, leading to strain localization during deformation. However, the PR values are larger in the case of 10^9^/s, indicating that higher strain rates facilitate the participation of more atoms in deformation, leading to a less significant degree of strain localization and a more homogeneous deformation of the glassy samples.

### 3.3. Correlation between Atomic Structure and Mechanical Response

**Figure 7:**
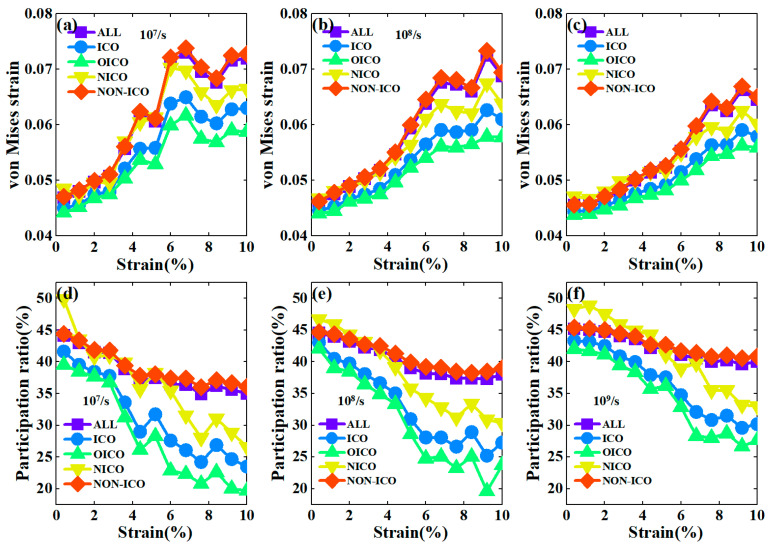
The average atomic von Mises strain (**a**–**c**) and PR (**b**–**f**) for various atomic clusters with strain rates of 10^7^/s, 10^8^/s, and 10^9^/s, respectively.

The above results illustrate the structure evolution and the mechanical response of CuZr metallic glass under tension at different strain rates. It is found that the strain rate has little impact on the structural evolution, but it significantly affects the mechanical response seen during deformation. It is not clear how local atomic structures respond to the deformation at different strain rates. Next, we analyzed the correlation between local atomic structures and the mechanical response during deformation and compared the von Mises strains for various atomic clusters, i.e., icosahedral clusters (ICO), including OICO and NICO, and non-icosahedral ones (NON-ICO). Here, $\eta_{i}^{Mises}$ was calculated for atom *i* in the configuration at the strain of ε by comparing it to the configuration at the strain of ε+Δε, with  Δε=1%. Figure 7a–c show the average von Mises strain for ICO, OICO, NICO, and NON-ICO atoms during deformation with strain rates of 10^7^/s, 10^8^/s, and 10^9^/s, respectively. It can be seen that the NON-ICO atoms have the largest von Mises strain, while the smallest strain is found for the OICO atoms whose $\eta^{Mises}$ values are smaller than those of ICO atoms. Moreover, $\eta^{Mises}$ values of NICOs differ greatly from those of OICOs, and are even comparable with those of NON-ICOs. Figure 7a shows that the difference in $\eta^{Mises}$ between these clusters is the largest in the case of 10^7^/s, but the difference becomes smaller with an increasing strain rate, especially for icosahedral clusters. It can be seen that more icosahedral clusters tend to participate in deformation with increasing strain rate. This also implies that the mechanical deformation of metallic glasses upon loading becomes more and more homogeneous with increasing strain rate.

Additionally, we also analyzed the participation ratio for various types of atomic clusters to compare the deformation propensity of these clusters under different loading conditions [37]. Figure 7d–f show PR values for five types of clusters during deformation with strain rates of 10^7^/s, 10^8^/s, and 10^9^/s, respectively. It can be seen that the PR values of ICO clusters are smaller than those of NON-ICO clusters during deformation, indicating that the NON-ICO clusters show a greater tendency to participate in deformation than ICO clusters, which is consistent with the results of recent studies [28,38]. However, OICO clusters and NICO clusters exhibit quite different PR values. The PR value of OICO is lower than that of NICO and ICO clusters, indicating that OICO has the strongest resistance to deformation under tensile loading. Furthermore, the PR values of NICO are higher than those of ICO, and are even comparable to those of NON-ICO clusters during deformation at different strain rates. The difference in von Mises strain and participation ratios between the OICO and NICO may result from their different networks. The network of the OICO is more compact and more resistant to deformation, while the network of NICO is sparser and less resistant to deformation [37,39]. Therefore, the mechanical responses of these five types of clusters are very different during deformation. However, this difference in mechanical response for different clusters was reduced at higher strain rates, meaning that different clusters have similar mechanical responses at higher strain rates. This benefits the homogeneous deformation of metallic glasses under tension conditions.

Therefore, although the strain rate has little impact on the evolution of the structural fraction during deformation, it significantly influences the mechanical response under loading conditions. Increasing the strain rate promotes a similar response to stress among different structures of the system, which suppresses the heterogeneity of deformation and allows the system to bear greater stress, thereby enhancing the yield strength of the system. However, a decreasing strain rate can enhance the difference in the mechanical responses of the different clusters during deformation, which promotes more heterogeneous deformation, indicating that the plastic deformation of the system is concentrated in a localized region, while the rest of the system remains largely elastic. This favors the formation of a shear band and leads to a decrease in the yield strength of the system.

Note that, in our work, strain rates of 10^7^/s, 10^8^/s, and 10^9^/s were applied in the above simulations. Thus, the strain rates considered in our work were much higher than those applied in some experiments, such as 10^−3^/s and 10^−2^/s [11,16,17,18,19,20]. In a regime with a very low strain rate, the deformation behavior may be quite different from that observed in our work. For example, the yield strength of Zr-based metallic glass is found to decrease with an increase in strain rates from 10^−4^/s to 10^3^/s [16,17], which is worth studying in future research.

## 4. Conclusions

In summary, we carried out MD simulations for Cu_64_Zr_36_ metallic glass and studied the effect of the strain rate on mechanical behavior under tensile deformation. It is found that the strain rate mainly affects the yielding behavior of metallic glasses by changing the mechanical response during deformation. It is also found that the fraction evolution of different atomic structures is different under different strain rates, but that the difference in the atomic structure evolution is not significant. Increasing the strain rate can promote more atoms to participate in deformation, leading to more homogeneous deformation of the system and enhancing the yield strength. Thus, the mechanical response of the system may provide a general understanding of the effect of strain rate on yielding behavior.

## Data Availability

The original contributions presented in the study are included in the article/Supplementary Materials, further inquiries can be directed to the corresponding author.

## Supplementary Materials

The following supporting information can be downloaded at: https://www.mdpi.com/article/10.3390/ma17112507/s1, Figure S1: The spatial distribution of the atomic von Mises strain at the strain of 2%, 4% and 6% for strain rates of 10^7^/s (a1–a3), 10^8^/s (b1–b3) and 10^9^/s (c1–c3), respectively. Figure S2: The average cluster size formed by atoms with von Mises strains larger than 0.1 (a) and 0.3 (b) as a function of strain during deformation with various strain rates.
