# Supplementary material for: Effect of Strain Rate on Mechanical Deformation Behavior in CuZr Metallic Glass
Source: Materials (Basel). 2024 May 23;17(11):2507. doi: 10.3390/ma17112507 (PMC11172737; doi:10.3390/ma17112507)
Supplement: Supplementary file 1 [file materials-17-02507-s001.zip › materials-3011655-supplementary.pdf]

Supplementary Material for

Effect of Strain Rate on Mechanical Deformation Behavior in CuZr Metallic Glass

Beibei Fan [1] and Maozhi Li [1,2,*]

[1] Beijing Key Laboratory of Opto-Electronic Functional Materials & Micro-Nano Devices, Department of Physics, Renmin University of China, Beijing 100872, China

[2] Key Laboratory of Quantum State Construction and Manipulation (Ministry of Education), Renmin University of China, Beijing 100872, China

* Correspondence: maozhili@ruc.edu.cn

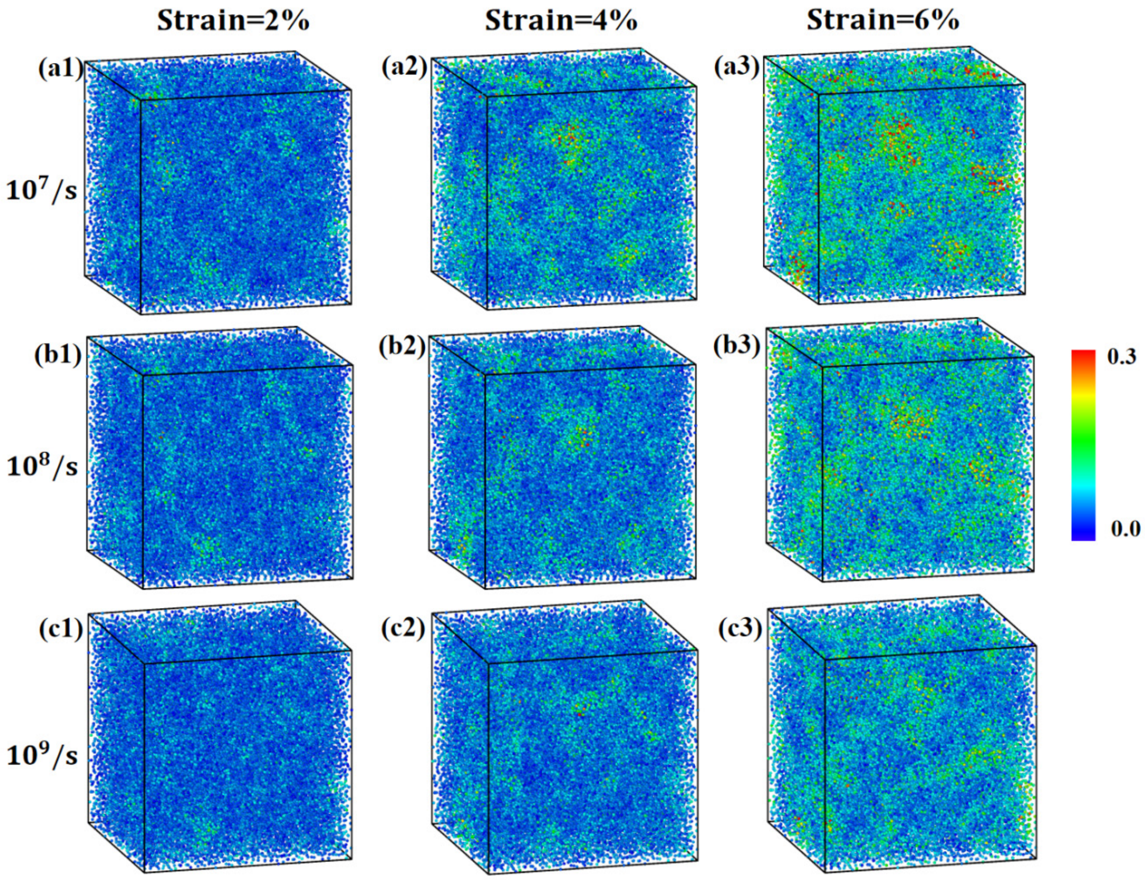

Figure S1 The spatial distribution of the atomic von Mises strain at the strain of 2%, 4% and 6% for strain rates of 10^7/s (a1-a3), 10^8/s (b1-b3) and 10^9/s (c1-c3), respectively.

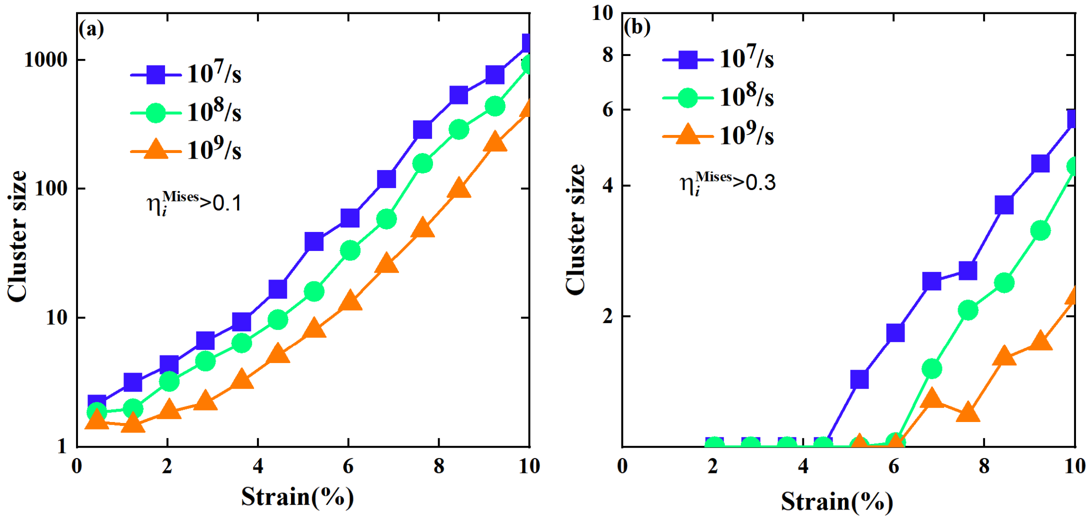

Figure S2 The average cluster size formed by atoms with von Mises strains larger than 0.1(a) and 0.3(b) as a function of strain during deformation with various strain rates.
